# Food-Based Social Enterprises and Asylum Seekers: The Food Justice Truck

**DOI:** 10.3390/nu10060756

**Published:** 2018-06-12

**Authors:** Fiona H. McKay, Kehla Lippi, Matthew Dunn, Bronte C. Haines, Rebecca Lindberg

**Affiliations:** 1 School of Health and Social Development, Faculty of Health, Deakin University, Geelong, VIC 3220, Australia; kehla.lippi@deakin.edu.au (K.L.); m.dunn@deakin.edu.au (M.D.); bronte.haines@deakin.edu.au (B.C.H.); 2 The Institute for Physical Activity and Nutrition (IPAN) and School of Exercise and Nutrition Sciences, Faculty of Health, Deakin University, Geelong, VIC 3220, Australia; r.lindberg@deakin.edu.au

**Keywords:** food security, social enterprise, asylum seeker, food aid, case study

## Abstract

People seeking asylum in high-income countries are vulnerable to food insecurity due to limited opportunities for social and economic participation. While charity organizations have long sought to provide food aid to those in need, the increasing number of people seeking this assistance requires alternatives. Using a case study approach, this research investigates The Food Justice Truck, which is a social enterprise designed to provide low cost, nutritious food to people seeking asylum with an aim to reduce the food insecurity burden. Twenty-seven people seeking asylum completed a structured interview (*n* = 15) or a semi-structured interview (*n* = 12). The majority of participants were female (*n* = 20) with an average age of 38.3 years (Standard Deviation (SD) 7.3; range 30–59) and over half were from Iran (*n* = 16, 59.2%) with most holding a temporary visa to stay in Australia (*n* = 15, 55.5%). Two key findings were identified including the fact that the FJT is at risk of creating and perpetuating a power imbalance. However, as a social setting, the FJT has the potential to promote and enable a social connection and create a positive experience. This research study adds valuable information to the literature by providing research on one alternative to traditional food aid. It was found that alternatives to traditional food aid may play a role in reducing the food security burden.

## 1. Introduction

Responding to social issues through creative or innovative means provides new ways to tackle both new and old problems. The social enterprise model has emerged from the non-profit sector and is characterized by its use of business solutions to solve social problems. Social enterprises are typically underpinned by a clear social purpose that can be addressed through trade in which profits or surplus are reinvested to fulfill a specific mission [1,2]. This approach recognizes the shortcomings of social support provided through government and charity and the limitations of the market economy in meeting public need. Given that social entrepreneurs tie their activities to actions deemed to have the greatest positive social impact, there is greater pressure for social enterprise to play a role in addressing health inequities [3,4]. Internationally, the positive economic benefits derived through social enterprises have been encouraged by governments with strong support for social enterprise in the United Kingdom (UK), Europe, United States of America (USA), South Korea, Malaysia, and Hong Kong [1].

Compared with these countries, social enterprise is relatively new and remains rare in Australia [1], contributing to less than 3% of the Australian gross domestic product [5]. Recent research on Australian social enterprises found that systematic barriers coupled with the largely paternalistic structure of the Australian charitable sector were key in stifling growth [6]. However, social enterprises that do exist within Australia have been shown to have a positive impact on health and well-being for individuals as well as communities with a range of enterprises supporting people with substance use problems, Aboriginal and Torres Strait Islanders, people living with a disability, people seeking asylum, and individuals experiencing food insecurity [7].

Food insecurity (the experience of inadequate or insecure access to nutritious food [8]) is a problem for an estimated 2 million Australians [9]. Vulnerable groups including those receiving welfare benefits, those experiencing precarious employment, and new migrant communities are at particular risk of food insecurity [9,10]. The increasing number of people seeking food aid (such as community meals as well as pantry and parcel programs) has put a strain on charitable organizations whose mandate it is to service this need. In 2017, Food Bank Australia reported a 10% increase in the number of people seeking assistance over the past year [9]. This increased demand has put pressure on hundreds of small-sized and medium-sized food relief agencies at a time when these providers are already struggling to meet the needs of their clients. As a result, many agencies have described turning people away or limiting the frequency in which clients can receive assistance [11].

There are currently around 30,000 asylum seekers living in Australia on temporary visas. Of these individuals, approximately 12,000 receive Status Resolution Support Services (SRSS) payments. This income support is provided at 89% of the job seeker allowance and is made available for asylum seekers while they wait for their claim to be finalized [12]. Asylum seekers receiving SRSS payments typically spend around half of their income on rent, one quarter on utilities, and much of the remaining on transport and essentials, leaving approximately $AUD 20 per week for food [13]. Such a limited income means that many are forced to skip meals or to rely on low-cost, nutritionally inadequate foods such as instant noodles and plain rice [14,15]. Financial barriers mean that asylum seekers in Australia are among those turning to charitable providers to meet their needs for food. The challenge for asylum seekers receiving SRSS is that, while this payment is below the poverty line and not sufficient to maintain living expenses [16], once granted, they are ineligible to receive food from some charitable providers [14], highlighting their particular vulnerability and the importance of alternative ways to access support.

While there are some who question the ability of the social enterprise to combat long-term food insecurity and to affect change within the broader food system [17], proponents of social enterprises argue that they provide an ‘additional tier of support’ in the food security space aside from the role of charitable organizations and government assistance [6,18,19]. This is supported by a large body of literature exploring the ability of social enterprises to address food insecurity internationally including the investigation of community supermarkets in Belgium [20] and the UK [18], food co-operatives-cum-enterprises in the UK [21], urban agriculture initiatives in New Zealand [22] and the USA [23], and food rescue in New Zealand [24]. Australia has a number of food-related social enterprises (for example, Food Ladder—www.foodladder.org). However, research on these enterprises is largely absent or small in scale [25].

The lack of independent research into the individual or community experiences of these enterprises presents a fundamental gap in the literature and leads scholars to question the value or applicability of social enterprises in Australia [6]. This current research seeks to address this gap by investigating the Food Justice Truck (FJT), a social enterprise designed to provide low cost, nutritious food to people seeking asylum with the aim of reducing the food insecurity burden. The aim of this research is to investigate the experiences of asylum seekers accessing this social enterprise.

## 2. Materials and Methods

This research employs an instrumental case study approach to explore the social enterprise based in Melbourne, Australia. This service provides fruits, vegetables, and other (mainly fresh) foods at low cost to people seeking asylum. An instrumental case study is one in which the researcher focuses on an issue of concern and then selects a bounded case to illustrate the issue [26]. This approach allows for an in-depth understanding of a particular case, which means that the case is unique in itself [27,28]. Given that people seeking asylum are often involved in time-consuming immigration processes, we provided two different opportunities for people to be involved in this research, which include structured and semi-structured interviews. Participants self-selected the method that was most suited to them. Ethics approval was gained from the Deakin University Human Ethics committee.

### 2.1. The Case

The Asylum Seeker Resource Center (ASRC) is the largest aid, employment, health, and advocacy organization for people seeking asylum in Australia. The ASRC is an independent non-government organization relying on community donations and philanthropy for 95% of funding [29]. More than 80 staff members and more than 1300 volunteers run the ASRC’s programs. Services are delivered to more than 2000 people seeking asylum at any one time through programs such as material aid, health care, legal aid, employment services, casework, and a food bank [29].

The FJT is a social enterprise initiative of the ASRC and it operates as a mobile fresh food market that provides ethically sourced and locally produced fresh fruit, vegetables, grains, pulses, bread, and tea to the public at market rates and to people who are seeking asylum at a discount. In response to studies highlighting high levels of food and nutrition insecurity in the asylum seeker populations in Australia [14,15,30,31], the FJT was created to provide people seeking asylum in Melbourne with an affordable option for buying nutritious food. While the ASRC has operated a food bank since they began, the FJT was designed to enable a greater reach in the community by operating in four different sites across the city. Unlike the onsite food bank, the FJT purchases foods from low-cost suppliers and operates less like a food charity and more like an enterprise. Affordability of the food supplied by the FJT was calculated based on the government payments that people seeking asylum receive, which includes $20 per week per family to spend on food [29]. Through the support of the general public who purchase goods at full price, the FJT retail model allows asylum seekers to purchase $80 worth of food for $20, which helps make a healthy and affordable diet more achievable for this population [29].

### 2.2. Recruitment

A variety of recruitment measures were employed. There were facilitated introductions by the FJT manager between the researchers and potential participants on site, approaching asylum seekers while they were shopping, a multilingual flyer at the register, and, lastly, snowball sampling. Structured interviews were conducted on site while semi-structured interviews were conducted either face-to-face at a convenient location or via telephone. All asylum seekers included in this research were over the age of 18 years.

### 2.3. Data Collection

To facilitate data collection with as many people seeking asylum as possible, this research utilized structured and semi-structured interviews. These approaches were conducted in separate phases with all attempts made to minimize any duplication in those completing both structured and semi-structured interviews.

Structured interviews took between 10 and 15 min to complete and consisted of approximately 50 questions (both open and close ended). Given their brevity and the largely qualitative nature of the questions, all structured interviews were conducted in English at FJT locations by student researchers on iPads using Qualtrics software. Participants who were not comfortable completing the interview in English were invited to participate in the semi-structured interviews at their convenience.

Semi-structured interviews were conducted using an interview guide by telephone or in-person in a private room close to the FJT. All semi-structured interviews were conducted by a trained student researcher. Interviews ranged from 25 min to 77 min and consisted of approximately 100 open and close-ended questions. Since we were seeking in-depth descriptions of the experiences of the users of the FJT, a government-operated telephone translation service was made available for these participants. The telephone service allows for simultaneous translation, which enables the researcher to immediately follow-up with the participant. Each interview was digitally recorded and transcribed verbatim. Interview participants received a $40 Coles-Myer voucher for their participation. Of the 12 participants interviewed, three took part in a follow-up interview via telephone to clarify or expand on previous statements and 7 participants utilized an interpreter.

Both the structured and semi-structured interviews contained a number of similar broad areas for investigation including:

1. Socioeconomic variables such as employment status, education attainment, visa status, and length of time in Australia. Information about age and postcode were also collected.
2. Food Justice Truck use, frequency of visits, and the types of food purchased, perceived food affordability, and estimated weekly food costs.
3. Experiences of the FJT.

In addition to expanding on the above questions and seeking more detailed answers, semi-structured interviews included a number of questions about food acquisition and preparation such as where participants shopped for food and why, what types of meals they prepared, and how their eating habits had changed compared to the home country. These questions were drawn from a range of studies that explored food insecurity for asylum seekers including McKay and Dunn [14], Nunnery and Dharod [32], and Piwowarczyk et al. [33]. To ensure appropriateness, the interview guide was piloted with a volunteer at the FJT who was also seeking asylum. Minor changes were made to improve the flow and comprehension of some questions, as a result of this pilot.

Field notes formed a final component of data collection for this study. These notes allowed for the collection of observation and reflection on actions and interactions [34]. Handwritten field notes were taken during visits to the FJT including notes on the set up of the FJT, the engagement between staff and customers, and the purchasing and shopping habits of the customers.

### 2.4. Data Analysis

Data from close-ended questions were analyzed using basic descriptive statistics to characterize the sample. Categorical data were reported using simple frequencies and percentages while continuous data were presented as means, medians, and standard deviations. Data were analyzed using Microsoft Excel 2016 (Microsoft, Redmond, WA, USA).

Open-ended data were analyzed thematically by hand, following the process described by Miles and Huberman [35]. Thematic analysis followed an inductive approach, which enabled the identification and generation of themes [36]. The constant comparative method in which data collection and analysis were conducted simultaneously, was employed. This allowed for the identification of patterns and ideas within the data. The transcripts were read and re-read to identify codes and categories from which themes were established. During data analysis, the research team met to discuss the identified themes including the properties and details of these themes. Observations captured in field notes have been used to supplement themes.

## 3. Results

In total, 27 asylum seekers completed a structured interview (*n* = 15) or a semi-structured interview (*n* = 12). The majority of participants were female (*n* = 20) with an average age of 38.3 years (SD 7.3; range 30–59). Around half of the participants were from Iran (*n* = 16, 59.2%) with a smaller number from Sri Lanka (*n* = 5), Iraq (*n* = 2), and one each from India, Malaysia, Pakistan, and Indonesia. Participants had been living in Australia (including time spent in immigration detention) for an average of 4.4 years (SD 1.5, range 1.5–10). Fifteen participants held a Bridging Visa E (*n* = 15, 55.5%). Sixteen (59%) participants had completed between 9 and 12 years of education and four (15%) had started or completed post-secondary qualifications. Three participants indicated that they were receiving income from work and 23 reported receiving income support in the form of SRSS payments. All participants were living in rented housing and most participants (*n* = 23, 85.1%) defined their housing as safe. The majority of participants (*n* = 26) reported having a working kitchen or some kitchen facilities.

Through the analysis of interview and observational data, two different experiences of the FJT were identified. It was found that the FJT is at risk of creating and perpetuating a power imbalance. However, as a social setting, the FJT has the potential to promote and enable social connection and create a positive experience.

### 3.1. Experience 1: Power-Relations at the Food Justice Truck

Through practices similar to those seen in the charitable food sector, the FJT employed a system of rationing and selectively displaying products. Typically, most in-season and abundant fruits and vegetables were made available for customers to self-select. However, some items, particularly those that were subject to predetermined quotas such as rice and watermelon, had to be requested by customers at the counter and were not on display. This was also the case for legumes, which were stored in bags inside the FJT and out of view. Through conversations with staff and volunteers, it was revealed that legumes had previously been displayed in dispensing self-service containers. However, this practice had ceased since the containers had proved problematic to set-up. This meant that, in order to purchase legumes, customers would need to know that the FJT stocked them and would need to ask a volunteer or staff member to access them on their behalf.

This research also identified a clear power imbalance between FJT staff and volunteers and asylum seekers accessing the service. This dynamic was largely due to the manner in which the FJT operated. The most conspicuous example of this was through the absence of price tags. While customers could verbally request the price of items, price tags (like those typically displayed in a supermarket) were not provided. Participants revealed that price tags were initially displayed when the FJT began operation. However, over the 12-month period prior to the study, the practice had ceased. While some participants felt comfortable asking volunteers or staff at the FJT for the price of items, others did not. While there was a price list available, not all customers were aware of it. The absence of price tags meant that prices could be subject to change or altered for different customers with some given a greater discount than others, which was largely at the discretion of staff. For customers who were comfortable requesting the price list or asking staff or volunteers for the price of foods, the absence of price tags did not pose a significant problem. However, for the majority of participants, the lack of price tags made it difficult to determine what to purchase. Some participants felt somewhat uncomfortable as a result of this practice. Instead of requesting that price tags be displayed, participants described alternative strategies for determining the cost of their shopping. Some participants noted the price of equivalent fruits and vegetables at other stores between FJT visits and made assumptions about the likely cost of foods at the FJT that week. Notably, many customers only discovered the total cost of their shopping once at the checkout. This was problematic for some customers who were observed returning items already selected once they learned the total cost of their shopping.

### 3.2. Experience 2: Social Connection and a Positive Experience

Despite the observed power imbalances, participants were positive about their experience of the FJT and the function of the FJT as a social enterprise. Participants were generally confident that they were getting a fair price for food at the FJT or at least the same price that they would find at a mainstream store. While some participants commented that there could be a greater variety of foods available or that they would like more availability of particular foods (for example, rice), there was also sentiment that the FJT was doing well in trying to cater to the range of needs present. For example, several participants mentioned how thankful they were that the FJT stocked watermelon and strawberries. These were seen as luxury items that would not be purchased at full price. Participants also discussed the measures staff and volunteers put in place to promote equality among customers. This included a standard start time for shopping so that all customers had the opportunity to access the full range of produce and no one could get there early and ‘cut the line’. There was also an acceptance that, while largely unspoken, the availability of some products was subject to change and needed to be rationed so that the majority of customers could share what was available.

Participants expressed gratitude that the FJT was created and existed especially for them. As such, for many participants, the FJT held value beyond that of simply a subsidized fruit and vegetable market and rather a space where they could be more comfortable. Furthermore, participants were aware that the FJT was largely funded by donations for the purpose of providing them with greater access to an increased variety of food and, as such, were disinclined to comment on its shortcomings. It was this sentiment that meant participants continued to access the FJT even though they could also access mainstream stores that often provided them with more consistency and a greater variety of produce.

Lastly, many participants valued the social environment that the FJT provided and described ways in which the staff and volunteers created an environment where they were treated with respect. Some participants had fostered friendships with staff and volunteers at the FJT and some even factored in the extra time it would take them to do their shopping due to time spent socializing. These friendships were most prominent for asylum seekers of Iranian decent and were more common among asylum seekers with some English language skills.

## 4. Discussion

This study sought to investigate user experiences of the FJT, a social enterprise aimed at providing low cost, nutritious food to people seeking asylum with an aim of reducing the food insecurity burden. Findings of this research suggest that the FJT has the potential to promote social connection and elicit positive experiences for customers. However, the power imbalance between asylum seekers and staff and volunteers contributed to negative experiences for some customers. There are two clear findings of this case study: (1) despite operating as a social enterprise, the FJT employed a number of practices more common to a food charity, which potentially undermines its aim to promote dignity, and (2) this food-based social enterprise may enable social connection for people seeking asylum.

The first key finding of this study is that, while the FJT operates as a social enterprise, in some ways, it also has the characteristics of charitable food aid. This included rationing of items, prices changing for different people, and insufficient provision of staple food items. These actions reproduce ideologies often associated with the charity sector. Research suggests that both stigma and shame are experienced by those receiving charity [37,38] including food charity [39,40]. As such, there is a concern that the actions of the FJT may result in, or reinforce, existing feelings of shame [41,42,43] or contribute to a sense of helplessness experienced by many asylum seekers in Australia [44]. This also has the potential to have negative ramifications for household food insecurity. A reliable, dignified, non-charitable nutritious source of food is required for individuals to achieve food security with the current FJT model unable to deliver on this. True achievement of food security includes more than just food access but encompasses stability of the food supply, personal choice, and having access to culturally-appropriate foods. Alternatives include The Stop Community Food Center in Ontario, Canada [45] and FoodLab in Detroit, Michigan, USA [46]. These community food programs are providing fresh and healthy foods at a cost-effective price for a range of people experiencing a socioeconomic disadvantage.

Importantly, thousands of people seeking asylum in Australia rely on charitable services for their essential requirements and, as a result, services are familiar with and seek to address the needs of people seeking asylum [14,47]. Many are well placed to do so and this work, although imperfect, saves lives and offers vital housing, food, legal, and material aid among other things. In addition, given that the social enterprise emerged from the non-profit sector [48], it is understandable that ambiguity or fluidity exists between these organizational structures and the way that they manage day-to-day activities. The ASRC operates largely as a non-profit organization with several smaller subsidiary social enterprises (in addition to the FJT, the ASRC also operates a cleaning and catering social enterprise), which potentially contributes to this ambiguity at the operational level of the FJT and the financial challenges associated with running the FJT and managing limited stock.

The second finding of this study is the potential for social enterprise not only to play a role in food relief but also to improve social connections among asylum seekers. Asylum seekers often experience loneliness and isolation from having arrived without family or support networks [49,50] with many describing feeling socially isolated from the broader Australian community [47]. The social role of the FJT is important since households with greater social support have been identified as less likely to experience food insecurity and experience greater overall well-being [51,52].

The networks that are created around programs like those presented in this case study can function as a source of information on community and government services. They can also support access to traditional foods and retailers, which are vital components of the local environment that assists new arrivals with feelings of belonging [53]. Other social enterprises working to alleviate food insecurity within the USA and Australia have demonstrated this capacity to increase social connection and provide an environment that can be free of shame or stigma with customers returning for social benefits such as community and societal acceptance and inclusion. Customers are treated with dignity and begin to make friends [25,54]. The current study found some people who are seeking asylum were using the FJT as a social space where friendships could be cultivated among asylum seekers, staff, and volunteers especially between individuals with proficiency in a common language.

## 5. Limitations

Despite the clear findings of this study, there are several limitations that need to be considered. The small, exploratory nature of this study, coupled with purposive sampling techniques means that there are perspectives of current FJT users that are not representative of all FJT users or people seeking asylum more broadly. However, by including both structured and semi-structured interviews and site observations across a variety of days and across multiple weeks, we have sought to sample as many opinions as possible. The use of interpreters in the interviews provides both methodological advantages and limitations in this research. While interpreters were essential to gain more detailed responses from participants, qualitative studies presenting data from interpreted interviews have been criticized for positioning the interpreter as a neutral, ‘invisible’ mouthpiece through which the interview takes place [55].

## 6. Conclusions

Until Australia changes immigration policies allowing appropriate financial and social support, asylum seekers living in the Australian community will remain at high risk of food insecurity. In the interim, targeted programs aimed at alleviating food insecurity must pursue the right to food in a manner that promotes equality. Although the social enterprise model of the FJT attempts to overcome the traditional hierarchical model of charitable food aid, it has been largely unsuccessful in this pursuit due to the power imbalance unwittingly reinforced via small but significant practices.

This research adds a valuable contribution to the literature by providing much needed research on one alternative to traditional food aid. It has also highlighted the need for a reflexive practice in alternatives to traditional food aid. Future initiatives should focus on strategies that promote dignity and equality and integrate the potential for social inclusion into their programs.
